# Current Trends and Challenges in Pediatric Access to Sensorless and Sensor-Based Upper Limb Exoskeletons

**DOI:** 10.3390/s21103561

**Published:** 2021-05-20

**Authors:** Guillaume Gaudet, Maxime Raison, Sofiane Achiche

**Affiliations:** 1 Department of Mechanical Engineering, Polytechnique Montréal, Montréal, QC H3T 1J4, Canada; maxime.raison@polymtl.ca (M.R.); sofiane.achiche@polymtl.ca (S.A.); 2 Marie-Enfant Rehabilitation Center, Research Center of Ste-Justine University Hospital Center, Montreal, QC H1T 1C9, Canada

**Keywords:** exoskeleton, upper limb, pediatrics, biomechatronics

## Abstract

Sensorless and sensor-based upper limb exoskeletons that enhance or support daily motor function are limited for children. This review presents the different needs in pediatrics and the latest trends when developing an upper limb exoskeleton and discusses future prospects to improve accessibility. First, the principal diagnoses in pediatrics and their respective challenge are presented. A total of 14 upper limb exoskeletons aimed for pediatric use were identified in the literature. The exoskeletons were then classified as sensorless or sensor-based, and categorized with respect to the application domain, the motorization solution, the targeted population(s), and the supported movement(s). The relative absence of upper limb exoskeleton in pediatrics is mainly due to the additional complexity required in order to adapt to children’s growth and answer their specific needs and usage. This review highlights that research should focus on sensor-based exoskeletons, which would benefit the majority of children by allowing easier adjustment to the children’s needs. Sensor-based exoskeletons are often the best solution for children to improve their participation in activities of daily living and limit cognitive, social, and motor impairments during their development.

## 1. Introduction

There is a wide variety of diagnoses that impair the arm movements of children, such as muscular dystrophy [1], spinal muscular atrophy [2], cerebral palsy [3], arthrogryposis multiplex congenita [4], and brachial plexus palsy [5]. Despite the important differences in the origin of these diseases, they all share a similar symptom: muscular weakness or stiffness at the upper limb. Such symptoms prevent these children from moving their upper limb freely. These diagnoses are further detailed in Section 2.2.

For children, the difficulty of moving the upper limb and, hence, interacting with their environment can have great consequences for the learning process. Indeed, it is known that children with limited exploration ability are at higher risk of developing cognitive, social, and motor impairments [6]. Moreover, weakness or impairments at the upper limb decrease autonomy in most activities of daily living (ADL), such as eating, bathing, getting dressed, and playing [7,8].

Traditional interventions to improve upper extremity function in children consist of strength training and aquatic therapy [9,10,11]. There is also evidence about the benefits of neuromuscular electrical stimulation to improve upper extremity strength, range of motion, and function [12]. The efficiency of these interventions relies on the frequency at which they are provided. However, it is often impossible for the specialized therapists to ensure sufficient hours to every child in order to maximize the intervention benefits. Another factor that ensures efficiency and security of these interventions comes from the patient feedback, which is not always reliable with children.

In the last few years, numerous sensorless and sensor-based exoskeletons have been developed to improve the quality of life of people with impairments at the upper limb, by acting both for rehabilitation, i.e., enhancement of the motor function, and assistance, i.e., support of the motor function. Furthermore, exoskeletons showed potential to increase intensive therapy [13] and reduce the workload of the therapists [14]. However, the targeted population for these devices is mainly adults who are recovering from a stroke. These include the ARMIN III [15], the CADEN-7 [16], the CAREX [17], the ETS-MARSE [18], the IntelliArm [19], the RUPERT [20], and the SUEFUL-7 [21].

Despite the increasing number of rehabilitation and assistance exoskeletons developed for adults, the options available for children are limited [22,23]. This is mainly due to the fact that a simple scaling of adult devices to children’s size is not appropriate for safe use. Indeed, additional considerations towards children’s growth, usage, and muscle force must be made in the design process since this population is heterogenous [22]. Further developments are desired since it is known that a greater functional improvement can be reached in robot-assisted rehabilitation compared to traditional interventions [24].

In the last few years, numerous reviews on upper limb exoskeletons have been published. However, these reviews mostly addressed the trends and challenges of exoskeletons and robotic rehabilitation devices for adults [25,26,27,28,29,30,31]. The review by Falzarano et al. [23] focused on pediatric rehabilitation devices but did not include any assistance exoskeletons, which are sometimes the preferred option depending on the child’s diagnosis.

The aim of this study is to (1) identify the different needs for children with an upper limb impairment, (2) present the latest development trends in sensorless and sensor-based upper limb exoskeletons, and (3) discuss future prospects to meet the specific needs in pediatrics.

## 2. Upper Limb Biomechanics and Diagnoses in Pediatrics

An exoskeleton is a device that works as an external skeleton to support the user [32]. These devices generally consist of complex structures imitating the anatomy of the human skeleton to guarantee the alignment between the joint axis of the user and the device [33]. Misalignment of the exoskeleton joints with the human body leads to spurious forces or torques at the human–exoskeleton interface, which can lead to discomfort and injuries [34]. The anatomy of the upper limb makes it difficult to design exoskeletons that replicate the kinematics and dynamics to efficiently support the user in ADL.

### 2.1. Upper Limb Biomechanics

**Figure 1:**
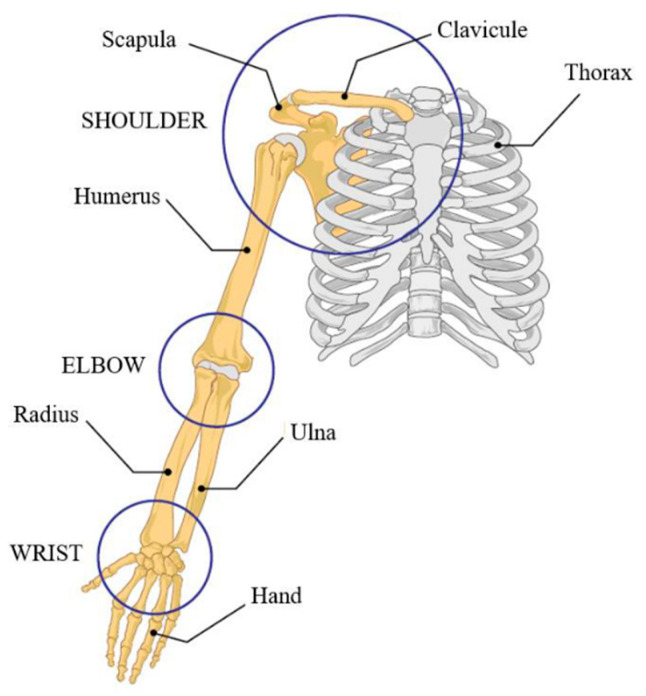
Anatomy of the upper limb.

The upper limb can be divided into four separate structures: shoulder complex, elbow complex, wrist joint, and fingers/hand (Figure 1). The shoulder allows for the largest range of motion of all human joints [35]. The shoulder complex (Figure 1) is composed of four articulations (glenohumeral, acromioclavicular, sternoclavicular, scapulothoracic) formed between three bones (clavicle, scapula, humerus). The simplest model of shoulder modeling consists of three degrees of freedom (DOF), which account for the three main movements of the shoulder, namely flexion–extension (FE), abduction–adduction (AA) as well as internal–external rotation (IE). However, using this simplified representation in exoskeleton design leads to misalignment since it does not account for the instantaneous center of rotation of the shoulder that changes as the upper limb moves [25]. Therefore, more complex models could be considered when modeling the exoskeleton shoulder mechanism to take into account the dynamic nature of the shoulder joint [36].

The elbow complex (Figure 1) is composed of two joints (humeroradial, humeroulnar) between three bones (humerus, radius, ulna). The elbow joints allow for two movements, namely FE and pronation–supination (PS). To ensure the accuracy of elbow modeling and kinematic compatibility with the exoskeleton, the forearm should be considered as a closed-loop mechanism [37]. It is also important to consider the displacement of the axis of rotation of the FE in order to guarantee joint alignment with the exoskeleton [38].

The wrist (Figure 1) allows for two movements, namely FE and radial–ulnar deviation (RU). The radiocarpal articulation is responsible for these movements. The rotation axes of FE and RU can be assumed coincident [26], which simplifies the design of mechanisms for exoskeleton wrist joints. Finally, the hand and fingers allow for grasping and dexterity through FE of all fingers and AA of the thumb.

### 2.2. Diagnoses in Pediatrics

There are several diagnoses in pediatrics that could benefit from sensorless or sensor-based exoskeletons. Although each one has certain specific characteristics, the common symptom is weakness in the upper limb that makes it difficult to move freely one or more DOF, as presented in Section 2.1. There exist too many diagnoses to cover them all in this review; therefore, only the most prevalent in the literature will be addressed.

#### 2.2.1. Cerebral Palsy

With a prevalence of 2.5 per 1000 children [39], cerebral palsy (CP) is the most common cause of physical disability in childhood. CP can lead to progressive deterioration of motor physical function, which can cause dependency and loss of productivity. CP is a lifelong condition and most patients will have a normal life expectancy [39].

There are multiple ways to classify CP, but in regard to upper limb function, the Manual Ability Classification System (MACS) is the most widely used by experts [40]. A MACS score of 2 is associated with reduced speed or quality of handling objects. For these children, active support of the upper limb might not be helpful [41]. However, they could benefit from gravity compensation at shoulder and elbow level to improve their motor function. The level of impairment becomes worse as the MACS score increases (maximum score of 5).

#### 2.2.2. Muscular Dystrophy

There exist a variety of types of muscular dystrophy, each affecting specific muscle groups, and with signs and symptoms appearing at different ages. The most common type of muscular dystrophy is Duchenne muscular dystrophy (DMD). Being an X chromosome-linked recessive disease, it mostly affects boys, with a prevalence of 1 in 5000 male live births [42].

Children with DMD will generally experience upper limb deterioration during their teens [1], which will greatly limit their autonomy in ADL. The use of exoskeletons in people with DMD could allow them to maintain social participation as well as independence in ADL by supporting every joint impaired by the disease.

#### 2.2.3. Spinal Muscular Atrophy

Spinal muscular atrophy (SMA) is an inherited autosomal recessive neuromuscular disorder, which results in the loss of motor neurons. Motor neurons are responsible for communication between the brain and the skeletal muscles. Without them, the communication is severed, which leads to muscle atrophy and weakness. The estimated prevalence of SMA is around 1 per 10,000 live births [43].

Children and adults with SMA experience progressive proximal muscle weakness and paralysis. Regarding the upper limb, the shoulder muscles are the most affected, impacting most of ADL. Assistance through gravity compensation could help them to increase their autonomy and independence.

#### 2.2.4. Arthrogryposis Multiplex Congenita

Arthrogryposis Multiplex Congenita (AMC) is not a specific diagnosis, but rather a clinical finding. AMC is used to describe a variety of conditions involving multiple congenital contractures that affect two or more different areas of the body. A contracture is a condition where the range of motion of a joint is limited. The prevalence of AMC is 1 in 3000 live births [4].

Most contractures in AMC take place in the shoulder, elbow, wrist, and hand, which limits greatly the ability to perform ADL. While there is no cure for AMC, occupational and physical therapy can help to improve the range of motion of contractures. In some cases, surgical procedures can be realized to improve the range of motion. Nevertheless, people with AMC could benefit from exoskeletons to improve the functionality of their upper limb by targeting the joints affected by contractures.

#### 2.2.5. Brachial Plexus Palsy

Brachial plexus palsy (BPP) is an injury of the brachial plexus nerves that occurs during labor. The estimated incidence rate of BPP varies between 0.1 and 8.1 per 1000 live births [5]. BPP injury typically occurs as the result of a lateral traction on the newborn’s head during the delivery process. The severity of nerve involvement can vary greatly among patients despite a similar mechanism of injury.

While most infants will recover spontaneously from BPP, around 35% of them will experience lifelong residual shoulder weakness, contracture, or joint deformity [44]. These impairments will negatively impact their autonomy in various ADL but could be limited with the use of an exoskeleton.

#### 2.2.6. Transitioning to Adulthood

It is important to acknowledge that the pediatric and adult health care systems are structured differently, and that the transition to the latter occurs at around 18 or 19 years old [45]. The process of transitioning from pediatric to adult health care is challenging and can negatively impact the quality of life of children with a disorder. One of the problems is that the adult health care system does not provide the same type of services as the pediatric system. For example, while pediatric care is often a combination of multidisciplinary teams and one-stop services, this is rarely the case in adult care [45]. There is also less expertise and/or interest in chronic illness of childhood within the adult sector [46].

Nowadays, most children with one of the diagnoses presented in this section will survive into adulthood. For CP, 65% to 90% of children will reach adulthood [47]. Life expectancy remains shorter for people with DMD, but with recent medical advances, they can live beyond childhood into their 30s and 40s [48]. For SMA, life expectancy varies from 2 years old to a normal lifespan based on the phenotype [49,50]. While some conditions involving AMC can have a higher mortality rate during infancy, life expectancy is normal past childhood [51]. Finally, BPP does not seem to have an impact on lifespan [52]. Therefore, it is important that children with an upper limb impairment have access to appropriate tools (e.g., exoskeletons) to limit cognitive, social, and motor impairments during their development.

## 3. Classification of Sensorless and Sensor-Based Upper Limb Exoskeletons

In this review, the term sensorless describes exoskeletons that do not contain any form of sensor. Conversely, the term sensor-based is used to describe exoskeletons that contain at least one type of sensor which can provide useful information to the user or therapist.

**Figure 2:**
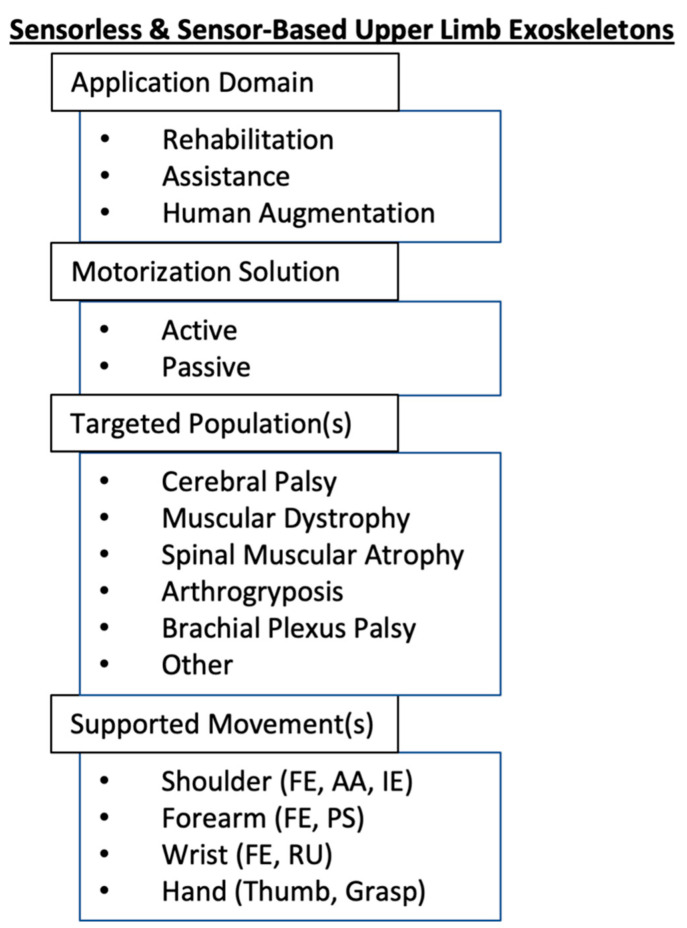
Categorization of sensorless and sensor-based upper limb exoskeletons in pediatrics. Abbreviations: FE: flexion–extension, AA: abduction–adduction, IE: internal–external rotation, PS: pronation–supination, RU: radial–ulnar deviation.

Upper limb exoskeletons can be classified using several methods [26], such as the applied segment, the number of active DOF, the method of actuation, the method of power transmission, and the application domain. In this review, the upper limb exoskeletons are first classified as sensorless or sensor-based. Each exoskeleton is then categorized regarding its application domain, motorization solution, targeted population(s), and supported movement(s) (Figure 2).

Each exoskeleton was first categorized according to its application domain: rehabilitation or assistance. A rehabilitation exoskeleton’s primary purpose is to enhance motor function by allowing partial or full recovery of the impairment. An assistance exoskeleton’s primary purpose is to support the motor function by facilitating the movements of the upper limb. While assistance exoskeletons can also have a rehabilitation purpose, the targeted population for this type of device is mainly people with a condition that cannot be improved. A third category, known as augmentation exoskeletons, was not included in this review. The primary purpose of these exoskeletons is to improve human strength and endurance [53]. Therefore, the principal users of augmentation exoskeletons are healthy adults, which are not the subject of interest for this review.

Regarding the motorization solution, each device was categorized as active or passive. An active exoskeleton uses powered actuators to move the user joints. A passive exoskeleton generally uses gravity compensation mechanisms to reduce the effect of gravity on the user’s arm.

The five diagnoses presented in Section 2.2, namely CP, DMD, SMA, AMC, and BPP, were used to categorize the exoskeletons. A sixth category, named Other, was also used to represent diagnoses not described in Section 2, such as stroke, traumatic brain injury, and dystonia.

The movements supported by upper limb exoskeletons can either be at the shoulder, the elbow, the wrist, the hand, or a combination of these.

## 4. Upper Limb Exoskeleton in Pediatrics

Table 1 and Table 2, respectively, show sensorless and sensor-based upper limb exoskeletons available in pediatrics. Each table provides information on the exoskeleton name, the application domain, the motorization solution, the targeted population as well as the articulations and movements supported. The number of DOF and the type of sensor(s) is also presented for each exoskeleton.

### 4.1. Sensorless Exoskeletons

This section presents briefly the sensorless upper limb exoskeletons developed for pediatrics and listed in Table 1.

#### 4.1.1. Dynamic Orthosis

The Dynamic Orthosis [54] is a pseudoelastic device that exploits the nonlinear mechanical characteristics of NiTi-based alloys. The aim of the orthosis is to support the residual voluntary motion and stabilize the posture of a joint dynamically. The properties of the NiTi elements are customized to the patient’s needs following an evaluation by a physician. This customization helps to improve the range of motion of stiff joints such as the elbow, the wrist, or the hand. This passive assistance exoskeleton was first used for children with dystonia and is still in development.

#### 4.1.2. Elbow Flexion Assist Orthosis

The Elbow Flexion Assist Orthosis [55] is a 1 DOF exoskeleton to improve elbow flexion in ADL. This passive exoskeleton uses the force of springs, combined with a sliding joint, to aid the user in bringing his hand to his mouth. The assistance provided by the passive mechanism helps to improve the active elbow flexion of the user. The exoskeleton was tested on one subject with AMC and could be beneficial for other diagnoses involving weakness at the elbow.

#### 4.1.3. Playskin Lift

Playskin Lift [56] is an exoskeletal garment that helps children to lift their arms against gravity. This passive exoskeleton consists of a long-sleeved garment equipped with casings under each arm, which house wire bundles. The diameter and number of wire bundles can be customized to match the support required by the user’s arm. Playskin Lift was tested on a 23-month-old infant with AMC and allowed 90 degrees of shoulder flexion at rest without limiting other movements. The garment can be used by children up to 3 years old.

#### 4.1.4. Playskin Air

Playskin Air [57] is a soft pneumatic exoskeleton for children that allows them to independently raise and lower their arms. This actuated soft exoskeleton was built according to the research on the Playskin Lift to extend its use to an older population. The actuation is controlled by a pushbutton located at the index. Playskin Air allows up to 120 degrees of shoulder flexion.

#### 4.1.5. Wilmington Robotic EXoskeleton (WREX) and P-WREX+

The WREX [59] and P-WREX+ [58] are passive assistance exoskeletons that provide gravity compensation at the arm and forearm, allowing users to move the upper limbs freely in 3D space. P-WREX+ was designed for toddlers (3–8 months old), while the WREX is aimed at children older than 1 year old. The gravity compensation is provided by elastic elements such as rubber bands or springs. These elements are customized to the user’s arm weight to provide gravity-eliminated movements. Passive joints at the shoulder allow for optimal placement and orientation of the exoskeleton. The use of WREX has been shown to be beneficial for children with DMD, SMA, AMC, and CP.

### 4.2. Sensor-Based Exoskeletons

This section briefly presents the sensor-based upper limb exoskeletons developed for pediatrics and listed in Table 2.

#### 4.2.1. Armeo Spring

Armeo Spring [61,62] is a 6 DOF upper limb exoskeleton that attaches to the patient’s upper and lower arm. With 3 DOF at the shoulder, 1 at the elbow, 1 at the forearm, and 1 at the wrist, this passive exoskeleton allows arm support and guidance through an adjustable spring mechanism. The Armeo Spring’s goal is to increase muscle strength and range of motion in different joints, in order to improve motor function. The position sensors in the Armeo Spring allow measurement of the patient’s performance, making it easier to adjust the support to reach the therapy’s objectives. The pressure sensors at the handgrip allow evaluation of graded grasp and release exercises. Multiple diagnoses could benefit therapy with this exoskeleton—for example CP, stroke, traumatic brain injury, and multiple sclerosis. The Armeo Spring is mainly used for in-clinic therapy.

#### 4.2.2. ChARMin

ChARMin [41] is a 6 DOF upper limb exoskeleton that was specifically designed for pediatrics. The exoskeleton has 3 DOF at the shoulder, 1 at the elbow, 1 at the forearm, and 1 at the wrist. ChARMin was built with a modular approach to cover different arm sizes. The proximal module is equal for all children aged 5 years and older. The distal module comes in two different sizes: a small one for children aged 5–13 and a large one for children aged 13 and older. This active exoskeleton provides single and multiple joint support during arm movements, with the objective of increasing strength and improving the quality of arm movements in ADL. ChARMin is intended for children with impaired arm motor function, such as CP.

#### 4.2.3. CT-DEA-Based Exoskeleton

The CT-DEA-based exoskeleton [64] is still in development. This early proof of concept has 1 active DOF for supporting the elbow FE. This exoskeleton uses dielectric elastomer actuators (DEA). The current prototype was tested on a human arm phantom model. While the velocity generated by the actuators is interesting, the range of motion is currently limited. The aim of this research project is to develop an active pediatric upper extremity exoskeleton made of soft materials.

#### 4.2.4. Hand Exoskeleton

The Hand exoskeleton [65] is a 2 DOF exoskeleton that assists hand opening. It has 1 active DOF responsible for opening digits 2–5 and 1 passive DOF responsible for following the natural AA during finger gestures. The developed mechanism passively follows the finger during the closing gesture. The exoskeleton can be easily adaptable to the user’s hand dimensions and is built by 3D-printing. This allows rapid modification of the exoskeleton as soon as the user outgrows it.

#### 4.2.5. IOTA

The isolated orthosis for thumb actuation (IOTA) [66] is a 2 DOF thumb exoskeleton developed for pediatric hand rehabilitation. It targets children with thumb in palm deformity, which is common in CP. This active exoskeleton allows the carpometacarpal and metacarpalphalangeal joints to move through ranges of motion required for ADL. IOTA is seen as an at-home rehabilitation exoskeleton that will complement in-clinic therapy. Different training modes are available, and a bend sensor at the wrist allows users with poor grasp control to actuate the orthosis to their will.

#### 4.2.6. MyoPal

MyoPal [67] is a commercial exoskeleton still in development by Myomo. It is the children’s version of the currently available MyoPro, a 2 DOF exoskeleton that assists in elbow FE and hand grasping. The exoskeleton is controlled by electromyography sensors placed on both the arm and forearm fixations. These sensors detect the user’s movement intention and then assist with that motion by providing sufficient actuation.

#### 4.2.7. PEXO

PEXO [68] is a 3 DOF wearable hand exoskeleton that was specifically designed for children aged between 6 and 12. The exoskeleton has 2 actuated DOF and 1 passive DOF. The actuated DOF allow, respectively, for FE of digits 2–5 and FE of the thumb. The passive DOF allows for thumb opposition/reposition. PEXO can be either controlled by simple pushbuttons or by an electromyography module. PEXO is intended to be an at-home training device to use in complement to conventional training to increase the rehabilitative effects. PEXO with the electromyography module could also be used as an assistance exoskeleton for children with permanent impairments.

#### 4.2.8. Soft Exoskeleton

This exoskeleton [69] is a soft robotic wearable for assisting and training upper extremity movements in infants. The exoskeleton has 4 active DOF: 1 at each shoulder (AA) and 1 at each elbow (FE). The wearable was tested on a human mannikin based on an average 12-month-old infant and showed great efficacy for assisting reaching tasks involving both the shoulder and elbow joints. Ongoing developments of the Soft Exoskeleton aim to integrate sensors, such as EMG, so that the device can adapt to changes in real time.

### 4.3. Current Trends

**Figure 3:**
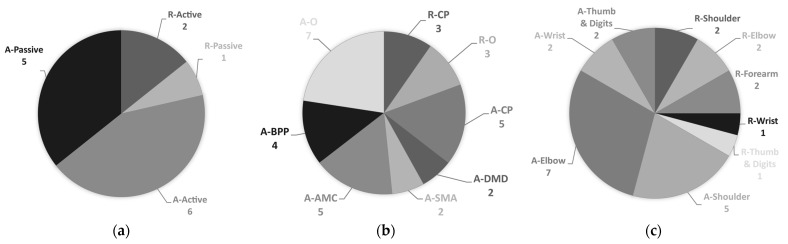
(**a**) Motorization solution (active or passive) of rehabilitation (R) and assistance (A) exoskeletons available in pediatrics; (**b**) Targeted population of rehabilitation (R) and assistance (A) exoskeleton in pediatrics. Abbreviations: CP: cerebral palsy, DMD: Duchenne muscular dystrophy, SMA: spinal muscular atrophy, AMC: arthrogryposis, BPP: brachial plexus palsy, O: other population; (**c**) Supported movements of rehabilitation (R) and assistance (A) exoskeletons in pediatrics.

A total of 14 different upper limb exoskeletons were identified for pediatric purposes. Of these exoskeletons, six of them are sensorless (Table 1) while eight are sensor-based (Table 2). Sensorless exoskeletons were all developed for assistance purposes. Five sensor-based exoskeletons were also developed for assistance purposes while the other three were aimed at rehabilitation. Out of the 14 exoskeletons identified, eight are active while six are passive (Figure 3a). Comparing the motorization solution with respect to the application domain, there are, respectively, two and six active rehabilitation and assistance exoskeletons, while there are, respectively, one and five passive rehabilitation and assistance exoskeletons. The active exoskeletons are motorized by either electric actuators (5) or soft actuators (3). The passive exoskeletons mostly use springs (4). There is also one assistance exoskeleton that uses shape memory alloys and another one that uses bundles of carbon wires.

Figure 3b shows the number of available exoskeletons for each targeted population with respect to the application domain. The main targeted population for all rehabilitation exoskeletons is CP (3). The three rehabilitation exoskeletons identified can also be used by other diagnoses in pediatrics, such as traumatic brain injury or pediatric stroke. For assistance exoskeletons, there is more diversity. CP, DMD, SMA, AMC, BPP, and other diagnoses were targeted by, respectively, five (5), two (2), two (2), five (5), four (4), and seven (7) different exoskeletons.

Figure 3c shows the number of available exoskeletons for each movement supported with respect to the application domain. Out of the three rehabilitation exoskeletons identified, two (2) exoskeletons target the shoulder, the elbow, and/or the forearm movements while one (1) targets the wrist and/or the hand. Assistance exoskeletons mainly target the proximal joints, with, respectively, seven (7) and five (5) devices for shoulder and elbow movements. There are two (2) exoskeletons for both wrist and hand movements.

## 5. Discussion

### 5.1. Rehabilitation Exoskeletons

To assist therapists and reduce their workload, robots are increasingly used in pediatric rehabilitation to support frequent and long training sessions. Indeed, rehabilitation with exoskeletons has multiple advantages over traditional interventions with a therapist.

First, it allows for high-frequency and high-intensity sessions [66,70] with great consistency since the rehabilitation device will not experience fatigue as the therapist would during the intervention and throughout his day of work. This absence of fatigue ensures high repeatability and reliability of training sessions. It is also easier for an exoskeleton to accurately control the assistance or resistance levels [71] to match the patient’s need for each training exercise.

A second advantage is that robot-aided rehabilitation allows the therapist to quantitatively monitor the progress of the patient throughout the rehabilitation process [66,70]. Indeed, the different sensors on ChARMin and Armeo Spring exoskeletons can record detailed kinematics indices such as accuracy, smoothness, straightness, and reproducibility to analyze the quality of upper limb movements during training [72]. Since traditional interventions often lack quantitative methods for evaluation [62,70], this possibility to pass from descriptive observations (e.g., older children show better movements) to quantitative conclusions is a great advantage of rehabilitation devices. Such data allow the therapist to identify and adapt to changes in movements, kinematics, and forces [73,74].

It is important to note that reaching and grasping function for ADLs may be challenged in different ways due to impairment in shoulder complex, elbow flexors, and/or wrist complex [75]. Therefore, it can be beneficial to target one or more specific joints during training. This specificity is possible with the rehabilitation exoskeletons identified. Indeed, the joints of an exoskeleton normally match the ones of the patient. This makes it possible to apply precise motions and torques to each articulation, which allows single joints to be independently trained. Another excellent feature of exoskeletons is that they allow the performance of reaching movements in 3D space. This is important as it allows the implementation of task-based therapies for the children [56]. The rise of exoskeletons in rehabilitation is therefore of the utmost importance because task-based therapies have been shown to better improve the rehabilitation process [73].

One drawback of rehabilitation exoskeletons, such as Armeo Spring and ChARMin, is that they do not allow at-home therapy since they are bulky and impossible to transport out of the rehabilitation center. Moreover, the high cost of rehabilitation exoskeletons makes it impossible for families to even consider buying one for home.

The obligation to pursue therapy at the rehabilitation center makes it difficult for children to have natural interactions with their environment, which can have a negative impact on their development. Indeed, for children, interaction with objects and other children is important in early language, perceptual–motor, social, and cognitive development and is a precursor for future essential life skills such as dressing and feeding [58]. While this is not a major limitation for children that can expect partial recovery of their motor function (e.g., CP), it is one for children that would benefit from continual assistance (e.g., AMC). For the latter, it is important to provide them with wearable solutions that allow them to improve their interactions in ADL.

In brief, rehabilitation devices bring great advantages to traditional interventions since (1) they do not suffer from fatigue, which allows for high-intensity and high-frequency training; (2) they provide reliable quantitative data to monitor the patient’s progress; (3) they allow for single joint training to adapt to patients’ needs, and (4) they allow for task-based therapies. Rehabilitation exoskeletons are generally sensor-based, which is essential to follow and adapt to the patient’s progress. However, rehabilitation exoskeletons (1) do not allow at-home therapy, (2) limit natural interactions because of their bulkiness, and (3) are not optimal for diagnoses without potential of motor function recovery.

### 5.2. Assistance Exoskeletons

As is the case for rehabilitation exoskeletons, assistance exoskeletons also allow for high-intensity and high-frequency training. The lower acquisition cost of these exoskeletons makes it possible to bring the device back home, hence reducing the travel time for therapy [66,70]. The lower cost is generally due to the lack or reduced number of sensors compared to rehabilitation exoskeletons.

The possibility of at-home therapy and using an assistance exoskeleton in ADL is of the utmost importance. Indeed, it is well accepted that the skills developed during in-clinic therapy (i.e., rehabilitation exoskeleton) do not always translate well into ADLs such as inserting a key into a door lock, combing hair, buttoning clothes, eating, and drinking [76].

The main objective of actual assistance exoskeletons is helping anti-gravity motions [60]. This is possible through mechanisms that compensate for the arm weight and support the arm during movements in space [77]. The continuous support that these exoskeletons allow makes them ideal for children who have diagnoses with upper limb impairments that cannot be improved, such as DMD, SMA, and AMC.

Although the wearability of the assistance exoskeletons is a great advantage over the rehabilitation exoskeletons, they are often limited in the number of supported movements. Indeed, there is currently no assistance exoskeleton that supports more than two movements, while some rehabilitation exoskeletons can support up to six movements. When designing an assistance exoskeleton, it is also important to make sure that it is light and compact enough so that it does not impact negatively other ADLs, such as leading to an unsteady gait [59] or reducing the ability to move easily [75].

An inconvenience of sensorless passive assistance exoskeletons is that it can be difficult to adjust the gravity compensation mechanism precisely to the children’s needs. Taking the WREX as an example, the final adjustments are made by adding or removing rubber bands. An incorrect number of bands will contribute to incorrect function and limit anti-gravity assistance [60]. Moreover, the parents will need to add or remove the rubber bands as their child gets older or depending on the ADL that he wants to perform. This lack of automatic adjustment can limit the acceptance of the exoskeleton for the user and families. Sensor-based exoskeletons such as the MyoPal do not have this limitation since the device is able to adapt to the user’s needs.

Another limitation of assistance exoskeletons is that they generally do not account for children’s growth. The focus on comfort, weight, and portability makes it difficult to design an exoskeleton that covers the range of patients from children to late adolescents [23].

### 5.3. Challenges for Pediatric Access

Research and development in upper limb exoskeletons in pediatrics are still recent. In comparison, the total number of exoskeletons for adults, based on the recent review of Gull et al. [25], is 69. Of the 69 exoskeletons for adults, there are 39, 23, and 7 exoskeletons developed, respectively, for rehabilitation, assistance, and human augmentation.

Despite the numerous options for adults, it is unfortunately not possible to simply scale them down in size [66]. The heterogenicity of children makes it difficult to have a one-size-fits-all exoskeleton. Indeed, the support needed in terms of joint torque by a 5-year-old child is not the same as a 13-year-old teenager, and both are significantly different from the one needed by an adult [78]. For example, most maximum upper limb joint torques of a 5-year-old child are below 10 N·m [79] while a 13-year-old can reach up to 40 N·m [80].

As stated by Aubin et al. [66], developing an exoskeleton in pediatrics “requires particularly careful design in terms of both its anthropometric size and weight”. The dimensions of the exoskeleton will vary greatly between a 5-year-old and a 13-year-old in comparison to adults. The pediatric exoskeleton ChARMin shows that a simple scale-down (i.e., ARMin [15]) can hardly adapt to this wide range of anthropometry. Indeed, a modular approach was chosen with ChARMin to allow the exchange of the distal part of the exoskeleton to satisfy the anthropometry of 5- to 18-year-old children.

Additional concerns towards the motivational aspect should be considered when designing an exoskeleton for children, since the level of motivation of children with an impairment during rehabilitation is generally difficult to maintain [81]. To incite active participation, the exoskeleton should be appealing and without any distractions [22].

Another challenge is that each diagnosis in pediatrics has specific requirements that give rise to different fitting, support, and control challenges, which require new technical methods and solutions [22]. The ideal exoskeleton for pediatrics will be able to adapt to different pathologies, adapt to children’s growth, and adapt to the assistance needed by the diagnosis. As stated by Hall and Lobo [56], it is also of the utmost importance that the assistance will be possible “without stigmatizing the user by negative social, psychological and cultural associations with disability”. Nevertheless, new methods to optimize the geometric and dynamic designs of exoskeletons are emerging [82] and will allow for lighter and smaller exoskeletons.

It is widely accepted that a user-centered design approach is preferred to guarantee better acceptability and continuous use of exoskeletons [83]. This design approach generally involves the user in both the initial and iterative stages of the devices’ development [84]. Users are able to provide important feedback through questionnaires, interviews, observations, and prototype testing [84]. However, children younger than 8 years old can be difficult to interview since they are still limited in its language development [85]. Therefore, the use of sensors in upper limb exoskeletons is of the utmost importance, since they can compensate for the lack of accurate feedback in children. Indeed, on-body sensors (e.g., inertial measurement units, force sensors, electromyography, etc.) allow the recording of useful in-body data, such as internal efforts, range of motion, and muscle forces, to enable the design of exoskeletons [86].

There exist a wide variety of sensors that can be integrated in pediatric exoskeletons. Nonetheless, the sensors used in upper limb exoskeletons should allow the measurement and processing of upper limb movement data [87] to adapt to the user’s needs during ADL [88] or to follow closely the rehabilitation progress [41]. Furthermore, sensors should also allow for device sensing, which can improve both the performance and safety of the exoskeletons [69]. For example, EMG and strain gauges can be used to predict the user’s motion intention [89], which could allow the actuation of the exoskeleton at the correct timing. Moreover, the recorded EMG can give information on muscle health, which can help to monitor muscle fatigue, reduce potential injuries, and improve the rehabilitation process [90]. Another example is the use of a load cell to measure the physical interaction between the exoskeleton and the user. Misalignment between the exoskeleton joints and the anatomical axes of rotation produces spurious forces and torques on the user, which causes discomfort and can lead to injury [91]. In pediatrics, joint misalignment can occur due to growth. The recording of spurious forces and torques could notify when a child outgrows its exoskeleton and point out the need for sizing adjustments.

## 6. Conclusions

The objective of this review was to present the principal diagnoses in pediatrics that causes upper limb impairments and identify the current trends and challenges for pediatric access to upper limb exoskeletons. The review highlighted that the most prevalent diagnoses in pediatrics do not allow for potential motor function improvements. Therefore, it is essential for these children to have access to an exoskeleton that can assist them in ADL. Assistance exoskeletons are better suited than rehabilitation exoskeletons in pediatrics. There exist both sensorless and sensor-based assistance exoskeletons. However, sensor-based exoskeletons are more promising since the additional data provided by the sensors allow better adjustment to the user’s needs. Nevertheless, the options in pediatrics are still limited when comparing to adults. This is mainly explained by additional challenges regarding children’s growth and wearability. New design methods, such as user-centered approaches, will help to tackle these challenges and improve the accessibility of pediatric exoskeletons. This is important to improve children’s participation in ADL and limit the risks of cognitive, social, and motor impairments during their development.

## Figures and Tables

**Table 1:** Sensorless upper limb exoskeletons in pediatrics.

Device Name	Application Domain	Motorization Solution	Targeted Population(s)	Degrees of Freedom	Supported Movement(s)	Company/Reference(s)
**Dynamic Orthosis**	Assistance	Passive–SMA	Other	3	Elbow–FE Wrist–FE Thumb and Digits–FE	Garavaglia et al. [54]
**Elbow Flexion Assist Orthosis**	Assistance	Passive–Springs	AMC	1	Elbow–FE	Wee et al. [55]
**Playskin Lift**	Assistance	Passive–Wire	AMC, BPP, Other	1	Shoulder–FE	Hall et Lobo [56]
**Playskin Air**	Assistance	Active–Soft	AMC, BPP, Other	1	Shoulder–AA	Li et al. [57]
**P-WREX+**	Assistance	Passive–Springs	AMC, BPP, Other	4	Shoulder–FE, AA Elbow–FE	Rahman et al. [58]
**WREX**	Assistance	Passive–Springs	DMD, AMC, CP, SMA	4	Shoulder–FE, AA Elbow–FE	Gunn et al. [59], Shank et al. [60]

Abbreviations: CP: cerebral palsy, DMD: Duchenne muscular dystrophy, AMC: arthrogryposis. SMA: spinal muscular atrophy, BPP: brachial plexus palsy, FE: flexion–extension, AA: abduction–adduction.

**Table 2:** Sensor-based upper limb exoskeletons in pediatrics.

Device Name	Application Domain	Motorization Solution	Type of Sensor(s)	Targeted Population(s)	Degrees of Freedom	Supported Movement(s)	**Company/Reference(s)**
**Armeo Spring**	Rehabilitation	Passive–Springs	Position, Pressure	CP, Other	6	Shoulder–FE, AA, IE Elbow–FE Forearm–PS Wrist–FE	Hocoma [61], Cimolin et al. [62], Peri et al. [63]
**ChARMin**	Rehabilitation	Active–Electric	Position	CP, Other	6	Shoulder–FE, AA, IE Elbow–FE Forearm–PS Wrist–FE	Keller et al. [13,22,41]
**CT-DEA-Based** **Exoskeleton**	Assistance	Active–Soft	Position, Force	N/A	1	Elbow–FE	Behboodi et al. [64]
**Hand** **Exoskeleton**	Assistance	Active–Electric	Position	CP	2	Digits–FE	Bianchi et al. [65]
**Isolated Orthosis for Thumb** **Actuation**	Rehabilitation	Active–Electric	Position, Bend	CP, Other	2	Thumb–FE, AA	Aubin et al. [66]
**MyoPal**	Assistance	Active–Electric	EMG, Position	BPP, CP, Other	2	Elbow–FE Thumb and Digits–FE	Myomo [67]
**PEXO**	Assistance	Active–Electric	EMG, Position	CP, Other	3	Thumb and Digits–FE	Bützer et al. [68]
**Soft** **Exoskeleton**	Assistance	Active–Soft	EMG	CP, Other	4	Shoulder–AA Elbow–FE	Kokkoni et al. [69]

Abbreviations: CP: cerebral palsy, DMD: Duchenne muscular dystrophy, AMC: arthrogryposis. SMA: spinal muscular atrophy, BPP: brachial plexus palsy, FE: flexion–extension, AA: abduction–adduction, PS: pronation–supination, IE: internal-external rotation, EMG: electromyography.

## Data Availability

Not applicable.
